# “Improving Diet Quality of Children with Dyslipidemia Who also Exhibit Picky Eating Behaviors”

**DOI:** 10.1007/s11883-024-01242-2

**Published:** 2024-09-26

**Authors:** Janet Carter

**Affiliations:** Medical University of South Carolina, Charleston, SC USA

**Keywords:** Dyslipidemia, Children, Picky Eating, Balanced diet, Heart-healthy Eating

## Abstract

**Purpose of the Review:**

This review is intended to serve as guidance for care providers working with children who have dyslipidemia and exhibit picky eating behaviors.

**Recent Findings:**

Picky eating behaviors in children can be very stressful for caregivers and children alike, even if they may not reach clinical significance. In the setting of lipid disorder treatment, picky eating can present an even greater challenge, since many of the foods considered most heart-healthy are not often considered “kid-friendly”.

**Summary:**

Care providers should validate caregivers’ concerns, screen for picky eating and be prepared to provide guidance to parents and a referral to a specialist, if needed. This review contains an itemized list of points to focus on with families and additional resources.

## Introduction

The impact of lifestyle habits on heart disease risk are well-established. Dietary intake plays a particularly important role in protection against cardiovascular disease. In children with disorders of lipid metabolism, adopting a healthy lifestyle is a critical component of their treatment plan. Picky or fussy eating behaviors are common in children with or without neurodevelopmental disorders, which can prove challenging and stressful for parents and children alike [1, 2]. Many well-established techniques for decreasing pickiness and improving the quality of a child’s diet have been outlined in behavioral literature. Providers can encourage families to integrate these techniques by providing guidance and support.

Reports of the prevalence of picky eating vary widely, from 5.6 to 54% with the discrepancy due to assessment methods, children’s ages, the countries where the studies were conducted, and other factors [3, 4]. Parents often report that concerns they raise during visits with care providers are not taken seriously [5]. While mild picky eating is common in childhood [2, 6, 7] and usually does not lead to nutrient deficiencies or growth concerns, it can cause stress in the family, affecting the parents and the child(ren) [1–3, 7–10]. There is mixed evidence for picky eating behaviors tracking into adulthood [2, 9], though an interesting study by Dial, et al. examined picky eating behaviors in young adults. In this study, most of the picky eaters ate fewer than ten foods, which lead to decreased intake of fiber, vegetables, and fruit, and increased incidence of anxiety and social phobia when eating around others [11]. Primary care providers are typically a family’s only sounding board for their concerns and receiving even a few recommendations or a referral to allied professionals can help tremendously. Taylor et al. outlined eight points that providers can emphasize during conversations with families who have concerns about picky eating behaviors [2]:

- Repeated exposure to new food.
- Use of non-food rewards.
- A positive approach to meals versus negative or pressuring.
- Role modeling, specifically eating vegetables and fruit and trying new foods.
- Limiting snacks and high-calorie beverages between meals.
- Realistic portion sizes for their child.
- Eating together with everyone eating the same food.
- Focusing on long-term goals and consistency.

The USDA “WIC Works Resource System” website is an excellent resource for providers and families and has a variety of educational handouts for families addressing picky eating. https://wicworks.fns.usda.gov/resources/picky-eaters.

## Picky Eating and a Heart-Healthy Diet

While picky eating isn’t clearly defined, there are many causes and treatment strategies outlined in the literature. For the purposes of this paper, we will use the definition of picky or “fussy” eating by Dovey, et al. “The consumption of an inadequate variety or quantity of foods through the rejection of a substantial amount of both familiar and unfamiliar foods” [12]. A description of picky eating proposed by Wolstenholme, et al. takes a more comprehensive look: “Fussy eating is an umbrella term describing the rejection of one or more food items, the limited intake or variety of foods, and/or frequent changes in food preferences due to novelty, sensory sensitivity, context/presentation of food, temperament/personality, age/developmental stage, and/or genetic and learned food preferences. Fussy eating can be expressed verbally or non-verbally (e.g. gestures, gagging, avoidance) and can [but does not always] have a perceived impact on the physical or psychological wellbeing of the child, parent, or family” [8]. For this discussion, we have excluded serious eating disorders like avoidant/restrictive food intake disorder [ARFID] and anorexia and the eating behaviors of children with neurodevelopmental disorders, as they are outside the scope. Below are the most cited causes of picky eating:

- Increased sensitivity to taste, smell, and texture [13], specifically a dislike of bitter tastes [6, 9].
- Early introduction of solids [13] or introducing solids after 10 months old [2, 6].
- Maternal depression or anxiety [13].
- Maternal picky eating behavior [13].
- Authoritarian and permissive parenting styles [6, 13, 14].
- Offering a reward for eating [13].
- Using food as a reward for behavior [8].
- Pressuring the child to eat [2, 8, 10, 13, 15].
- Unstructured or uninvolved parenting [13].
- TV/screen time during meals [13].
- Offering alternative options to the disliked food [8] or allowing children to decide what foods are offered [6].

Being able to recognize and assess picky eating is a bit challenging. Some of the most common behaviors of picky eaters include rejection of new foods, having a small range of liked foods, reduced intake of vegetables, reduced total intake of food, and general disinterest in food [1]. During an assessment, it is important to listen to the caregivers’ concerns while getting information about the frequency, duration, and severity of symptoms. There are some formal assessment tools available, but they may not be viable options for the clinic setting. It is also important for care providers to consider the underlying factors that increase the likelihood of picky eating, including neurodevelopmental disorders (specifically autism spectrum disorder and attention deficit hyperactivity disorder) [10, 16], neurologic abnormalities that affect swallowing, anxiety, depression, eating disorders, maternal overweight/obesity [10], maternal depressive symptoms, temperament of child and the parent-child relationship, and low income [2, 7, 9]. Providing help with resolution of these underlying factors may help improve the picky eating behaviors.

For children with disorders of lipid metabolism, adoption of a heart-healthy eating pattern is the foundation of their treatment plan. A heart-healthy eating pattern includes copious vegetables and fruit, lean meat and fatty fish, whole grains, legumes, and sources of unsaturated fat, such as avocado, nuts, seeds, and nut butters. It’s also important to have low intake of deep-fried food, foods high in saturated fats, sugary beverages, and other calorie-dense/low-nutrient snack food items. It is well established that picky eaters have lower intake of vegetables and fruit with additional evidence showing low intake of meat and seafood [3, 6]. One study also assessed intake of seeds, legumes, and whole grains which was found to be low in picky eaters [15]. Children with picky eating also tend to consume higher amounts of savory snacks, sweets, sugary drinks, and French fries [6, 10]. Broadening a child’s food repertoire takes time and effort. There is consensus in the literature about the best behavioral strategies for picky eating. Below are the most cited strategies:

- Caregiver behavior-focused (“Parent beliefs, emotions, and awareness should be targeted alongside parent feeding practices to increase effectiveness of interventions.”) [8].
  - Practice positive role modeling and eating with the child(ren) [2, 3, 13, 16, 17].
  - Set rules, such as “must try everything” or “must try a certain amount” [17].
  - Use an authoritative parenting style [14].
  - Have a responsive and autonomy-promoting parenting style [7, 13].
  - Set boundaries and provide encouragement [7, 14].
  - Use open communication [14].
  - Engage in problem solving with the child(ren) [14].
  - Practice supportive co-parenting [3].
  - Tolerate age-appropriate behaviors like getting dirty [19].
- Practice repeated exposure to rejected foods (8–10 exposures) [9, 13, 14, 18, 19], specifically without changing the presentation (cooking method, added sauces, hiding it in accepted food, etc.) [18].
- Have consistent times and locations of meals and snacks [19].
- Encourage multisensory play (touch, observe, taste) [14].
- Keep mealtimes stress-free, calm, and positive [19].
- Involve the child(ren) in food preparation [13, 19].
- Take child(ren)’s preferences into consideration [17].
- Increase physical activity [13].
- Follow the practice of caregiver providing the meal and children deciding whether to eat and how much to eat [8, 19, 20].
- Include stimulus control [16].
- Incorporate goal setting and self-monitoring [16].
- Promote self-efficacy and self-management skills [16].

One study by Anwar et al. examined nutrition supplementation in children with picky eating behavior and found significant improvements in growth, appetite, and intake of a more varied diet when supplementation in the form of a nutritional beverage was combined with diet counseling [4].

The strategies described to improve pickiness are effective, but it was noted in one study that they are not always consistently implemented in the home environment, despite parental knowledge of these strategies [18]. Specifically, caregivers, despite knowing the importance of repeated exposure to food, were not consistent in practicing this technique, likely driven by the child’s behavior and the parent’s desire to ensure that they eat. The cost of food and concerns about food waste were also factors leading to inconsistency [18]. It is important for care providers to recognize and address these barriers to improvement. Additionally, these strategies prove especially difficult to implement in the setting of neurodevelopmental disorders like autism or ADHD, specifically when these children experience food neophobia [18].

Another common theme in the literature is that caregivers perceive pickiness as more severe than it is [7]. This creates some challenges, as it has been noted in a qualitative study that parents believe their concerns are dismissed by care providers [5]. Parents expressed desire for “step-by-step” strategies to be provided [5]. Occupational therapy or Occupational Performance Coaching are excellent resources for caregivers of picky children and are effective in improving child eating behaviors and caregiver perception of the behaviors [21].

Picky eating is common in childhood and typically resolves on its own with little intervention though families look to their child’s provider(s) for guidance [2, 7]. There is little evidence that less-severe picky eating causes malnutrition or long-term negative health consequences [7, 10]. Some studies show that picky eaters are more likely to be underweight and have shorter stature [2–4, 6, 13, 14], others show greater risk of overweight in picky eaters [2–4, 9, 15], and still others show no relationship to body composition or BMI z-score [2, 7, 10]. There is a fair amount of evidence that picky eaters have lower intakes of vegetables and fruit [2, 6, 10, 15, 20], meat and fish [2, 10], zinc and iron [2, 6, 10, 22], and fiber [2, 22], but none of the studies reviewed here made mention of clinical manifestations of these lower intakes other than constipation [2–4, 6, 10, 22] and low serum zinc levels [23].

One interesting study looked at persistence of very picky eating [22]. Researchers found that picky eating behavior in young adults lead to similarly decreased consumption of vegetables, fruit, and dietary fiber as in children with picky eating behaviors [11]. Most of the subjects in the study ate fewer than 10 different foods and were more likely to report obsessive compulsive disorder, depressive symptoms and anxiety when eating with others [11]. This underscores the importance of addressing picky eating behaviors in young children when it typically presents and peaks (around 2–6 years) [15].

## Conclusion

In the absence of a neurodevelopmental disorder or physical abnormality, picky eating likely has little long-term consequences, but is a prevalent concern for caregivers, increasing caregiver anxiety and potentially resulting in adverse interactions between the child and the caregiver. Having uniform assessment tools and formal guidelines would be helpful for providers including primary care providers, occupational therapists, dietitians, etc [1]. Lipid treatment programs, including obesity treatment programs, should address picky eating when it presents [9]. Studies that have included interventions have shown success in improving the eating behaviors of picky eaters [14, 15].

Picky eating behaviors should not be ignored, and caregivers’ concerns should be validated by the child’s care team, but thankfully, these behaviors tend to resolve with education and behavior change. Care providers should always monitor picky eaters for signs of malnutrition or effects on growth. In the setting of the treatment of lipid disorders, the focus of improved intake should be on increasing intake of foods rich in unsaturated fats and decreasing intake of saturated fats. Given the evidence that supports the benefits of lifelong healthy eating, strategies to improve eating behaviors in children with dyslipidemia have the potential to significantly improve outcomes. In addition to education, caregivers require time, patience, and support to be successful.

## Data Availability

No datasets were generated or analysed during the current study.
